# Fabrication of Efficient Organic-Inorganic Perovskite Solar Cells in Ambient Air

**DOI:** 10.1186/s11671-018-2714-z

**Published:** 2018-09-21

**Authors:** Jian Wu, Jing-Jing Dong, Si-Xuan Chen, Hui-Ying Hao, Jie Xing, Hao Liu

**Affiliations:** School of Science, China University of Geosciences, Beijing, 100083 China

**Keywords:** Ambient air, Moisture, N-butyl amine, Perovskite solar cells, 81.05.Lg61.72.Cc81.10.Dn84.60.Jt

## Abstract

Although many groups have been trying to prepare perovskite solar cells (PSCs) in ambient air, the power conversion efficiency (PCE) is still low. Besides, the effect of moisture on the formation of perovskite films is still controversial. In this paper, we studied the effect of moisture on the formation of perovskite films in detail, and found that moisture can speed up the crystallizing process of PbI_2_ films to form poor-quality films with large grain size and surface roughness, while, for the conversion of PbI_2_ to perovskite films, a small amount of moisture is not adverse, and even beneficial. On this basis, we report the successful fabrication of efficient mesoporous PSCs with PCE of 16.00% under ambient air conditions at 25% relative humidity by adding a small amount of n-butyl amine into the solution of PbI_2_ to enhance the quality of PbI_2_ films and thus to achieve high-quality perovskite films with smooth surface, large crystal grains, and high crystal quality.

## Background

Organic-inorganic perovskite solar cells (PSCs) have become a pacemaker in the photovoltaic community with a rapid increase in the power conversion efficiency (PCE) from initial 3.8% in 2009 to a recently reported 22.7% [1–3], due to the high absorption coefficient, low exciton-binding energy, long charge carrier diffusion length, and high mobility of the organic-inorganic perovskite materials [4–12]. Unfortunately, the organic-inorganic perovskite materials are very sensitive to moisture due to the hygroscopic nature of the organic components [13], so the fabrication and long-term stability of PSCs in ambient air has been considered as one of the major challenges for future large-scale application. Interface engineering and encapsulation technology are widely used to improve the stability of PSCs in ambient air, which gains obvious effect [14, 15]. To avoid moisture in ambient air during the fabrication process of PSCs, most groups prepare PSCs inside a N_2_-filled glove box. There are also several research groups, who found that when perovskite materials were prepared in glovebox under inert atmosphere, the resulting perovskite films remained poorly crystalline, but, once exposed to a special controlled humidity atmosphere, a rapid crystallization into highly oriented crystallites was observed [16–19]. However, for future mass production, it is the best choice to fabricate highly efficient PSCs with a facile and simple way in ambient air, neither glove box nor a special controlled humidity atmosphere.

Recently, strategies have been achieved to develop the air-processed PSCs, and they can be generally divided into two methods: (i) finding unique and simple manufacturing processes to achieve high-quality perovskite films in ambient air; (ii) exploring new perovskite materials with fundamentally good air stability. For the first method, preheating substrates has been employed as a simple and effective manufacturing process to fabricate PSCs in ambient air. One group reported a highest PCE of 7.9% by preheating substrates at 200 °C before the one-step spin-coating of perovskite films in ambient air [20]. For the second method, CsPbBr_3_-based inorganic PSCs have been fabricated in ambient air which exhibits the highest PCE of 7.78% [21]. Besides, Tai et al. reported a type of efficient and stable perovskite solar cells prepared in ambient air by using lead (II) thiocyanate precursor instead of PbI_2_. Upon optimization, the devices demonstrated average PCEs over 13%, together with the maximum value of 15% [22]. However, the authors did not study the role moisture plays on the crystallizing process of PbI_2_ films and conversion of PbI_2_ to perovskite films in detail. Although many groups have been trying to prepare PSCs in ambient air, the PCE is still low, seldom reaches 16% as reported [13]. Besides, the effect of moisture on the formation of perovskite films is still controversial.

In this work, we report the successful fabrication of efficient mesoporous PSCs with PCE of 16.00% under ambient air conditions at 25% relative humidity (RH) by adding a small amount of n-butyl amine (BTA) into the solution of PbI_2_ to enhance the quality of PbI_2_ films and thus to achieve high-quality perovskite films with smooth surface, large crystal grains, and high crystal quality. Besides, to study the effect of moisture on the formation of perovskite films, the performance of mesoporous PSCs prepared under different RHs without BTA additive has been investigated in detail. Combining SEM and XRD characterizations of PbI_2_ and perovskite films with and without BTA additive, it is clear that moisture can speed up the crystallizing process of PbI_2_ films to form poor-quality films with large grain size and surface roughness, while, for the conversion of PbI_2_ to perovskite films, a small amount of moisture is not adverse, and even beneficial.

## Methods

### Fabrication of Perovskite Solar Cells

**Fig. 1:**
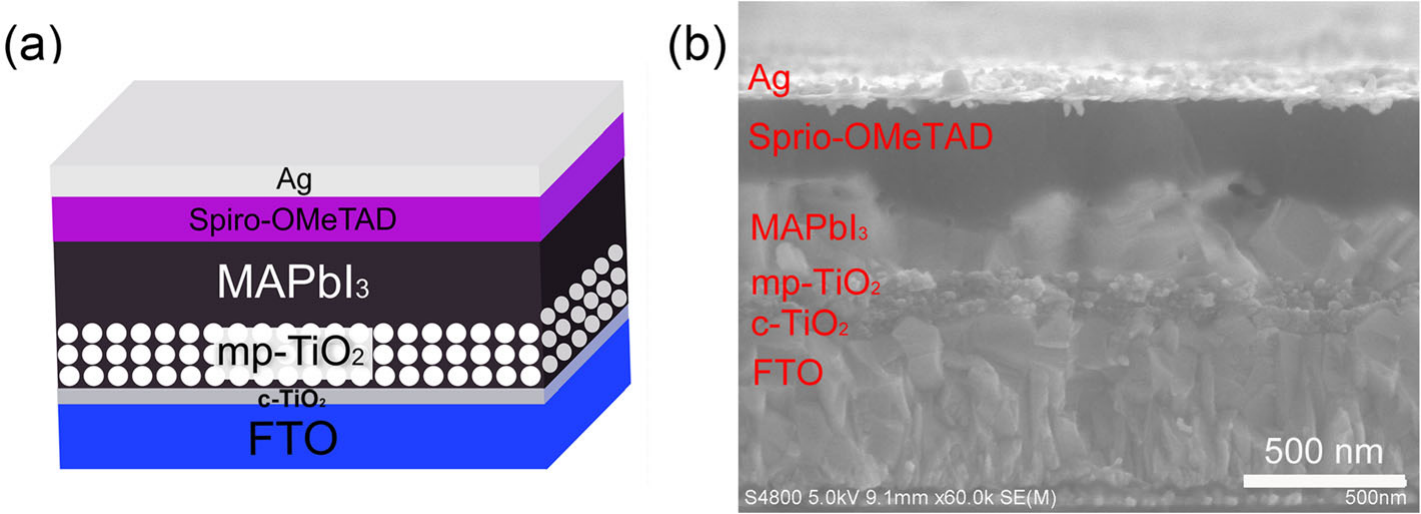
**a** A schematic diagram of the mesoporous PSCs. **b** The SEM cross-section image of the device, with the structure FTO/c-TiO_2_/mp-TiO_2_/MAPbI_3_/Spiro-OMeTAD/Ag

A mesoporous device structure was adopted for the device fabrication, as shown in Fig. 1a. Fluorine-doped transparent conducting SnO_2_-coated glass substrates (FTO) with a sheet resistance of 7 Ωsq^−1^ were cleaned with acetone, ethanol, isopropanol, deionized water, and isopropanol respectively. A compact TiO_2_ (c-TiO_2_) layer was deposited on the FTO substrates by spin coating at 3000 rpm for 30 s (repeat twice, followed by annealing at 150 °C for 15 min for each time), and then the c-TiO_2_ layer was annealed at 500 °C for 30 min in air. After cooling to the room temperature, a mesoporous TiO_2_ (mp-TiO_2_) layer was deposited by spin-coating at 5000 rpm for 45 s using a TiO_2_ paste (18NRD) diluted in EtOH (1:7, weight ratio). After drying at 80 °C for 40 min, the mp-TiO_2_ layer was sintered at 500 °C for 30 min. Once cooling to the room temperature, the film was dipped in the aqueous solution of TiCl_4_ for 30 min at 70 °C, rinsed with deionized water, and finally annealed at 500 °C for 30 min. After that, perovskite films were prepared by the two-step spin-coating method as follows. Firstly, 1 M of PbI_2_ in N,N-dimethylformamide (DMF) (adding a small amount of BTA into the solution) was spin coated onto the mp-TiO_2_ layer at 3000 rpm for 30 s, and then annealed at 70 °C for 15 min. After the PbI_2_ films cooled down to room temperature, the solution of methylammonium (MA) iodide was spin coated onto PbI_2_ films at 4000 rpm for 45 s. Finally, the samples were annealed at 100 °C for 30 min to grow into MAPbI_3_ films. Cooling down to room temperature, the 2,2′,7,7′-Tetrakis[N,N-di(4-methoxyphenyl)amino]-9,9′-spiro-bifluorene (Spiro-OMeTAD) layer was spin-coated at 2000 rpm for 45 s, where 80 mg Spiro-OMeTAD in 1 mL chlorobenzene solution was employed with the addition of 28.8 μL 4-tert-butylpyridine (TBP) and 17.7 μL lithium bis(trifluoromethanesulfonyl)imide (Li-TFSI) solution (520 mg Li-TFSI in 1 mL acetonitrile). Finally, Ag back electrode was deposited by thermal evaporation. The active area of the device was 0.1 cm^2^.

During the fabrication process, four solar cells were fabricated in each FTO substrate. Among which, if the maximum PCE deviation is less than 3% in at least three solar cells with higher PCE values, then their performance parameters will be recorded.

### Characterization

Current density-voltage (J-V) characteristic curves of photovoltaic cells were measured by a source meter (Keithley, 2400) with a solar simulator (Zolixss150) under 100 mW cm^−2^ AM 1.5G illumination; the light intensity was calibrated by means of a silicon reference solar cell. The active area of the devices was 0.1 cm^2^. Typical J-V curves were obtained from scanning in reverse bias direction at the step width of 200 mV. The voltage was scanned from 1.2 to − 0.2 V at a rate of 100 mV s^−1^. The J-V measurement was carried out in ambient air. An emission Hitachi S-4800 was used to obtain scanning electron microscopy (SEM) images with the electron beam acceleration in the range of 15 to 60 KV. The characteristic X-ray diffraction (XRD) patterns were recorded between 10° and 70°, using the Cu-Kα radiation at 1.5405 Å. The light absorbance spectra were required by using Cary 5000 UV-Vis spectrophotometer in the wavelength range of 200 to 1200 nm with 1 nm increment. All the measurements of these films were carried out in ambient air without humidity control.

## Results and Discussion

**Fig. 2:**
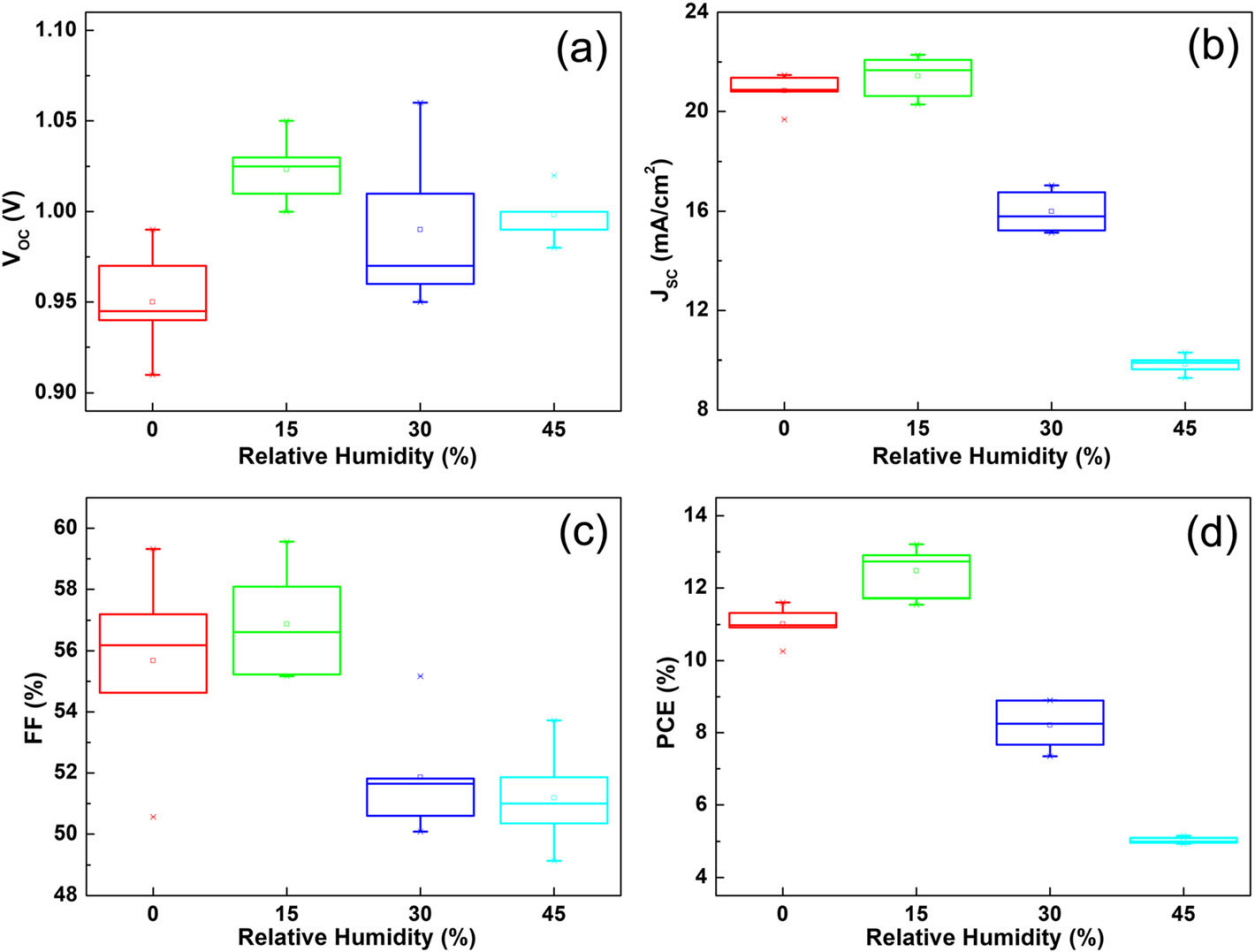
Box Charts of **a** V_OC_, **b** J_SC_, **c** FF, and **d** PCE for mesoporous PSCs prepared under different RHs at 30 °C without the adding of BTA during the two-step spin-coating of perovskite films

To study the effect of moisture on the formation of perovskite films, two-step spin-coating experiments under different RHs at 30 °C without the adding of BTA were designed, and the corresponding statistic results of detailed photovoltaic parameters are shown in Fig. 2. With the increase of RH from 0 to 15%, all of the photovoltaic parameters, including open-circuit voltage (V_OC_), short-circuit current density (J_SC_), fill factor (FF), and PCE, are improved obviously. As reported, a small amount of moisture could promote ion diffusion in the precursor film, facilitate the perovskite crystal growth, and thus induce a rapid crystallization into highly oriented crystallites [13, 23]. Therefore, better performance of PSCs under 15% RH was observed, compared with the PSCs fabricated in the glovebox (0% RH). Going on to increase the RH, the photovoltaic parameters, V_OC_, J_SC_, FF, and PCE, begin to drop sharply, as shown in Fig. 2. When the RH rises to 45%, the average value of V_OC_, J_SC_, FF, and PCE drops to 1.00 V, 9.84 mA/cm^2^, 51.02%, and 5.02%, respectively. The drastic decline of PCE under 45% RH is mainly caused by the sharp decrease in J_SC_. It was reported that too much moisture could cause poor surface morphology and even decomposition of perovskite films, so the J_SC_ of PSCs dropped sharply under 45% RH [18]. According to the results above, the optimal humidity conditions for two-step spin-coating of perovskite films in ambient air without the adding of BTA is 15% RH, and the corresponding highest PCE is 13.21% (average PCE is 12.48%), which is too low to meet future mass production. Besides, the results above are still insufficient to explain the role that moisture plays on the formation of perovskite films during two-step spin-coating.

To improve the performance of the PSCs prepared in ambient air and further investigate the role that moisture plays on the formation of perovskite films, a small amount of BTA was added into the solution of PbI_2_. BTA, who has strong volatility, well infiltrative property, and strong Lewis base nature [13], will isolate the PbI_2_ films from part of the moisture in ambient air, help the PbI_2_ solution to spread out on the substrates easily and homogeneously, and greatly slow down the crystallization rate to form high-quality PbI_2_ films.

**Fig. 3:**
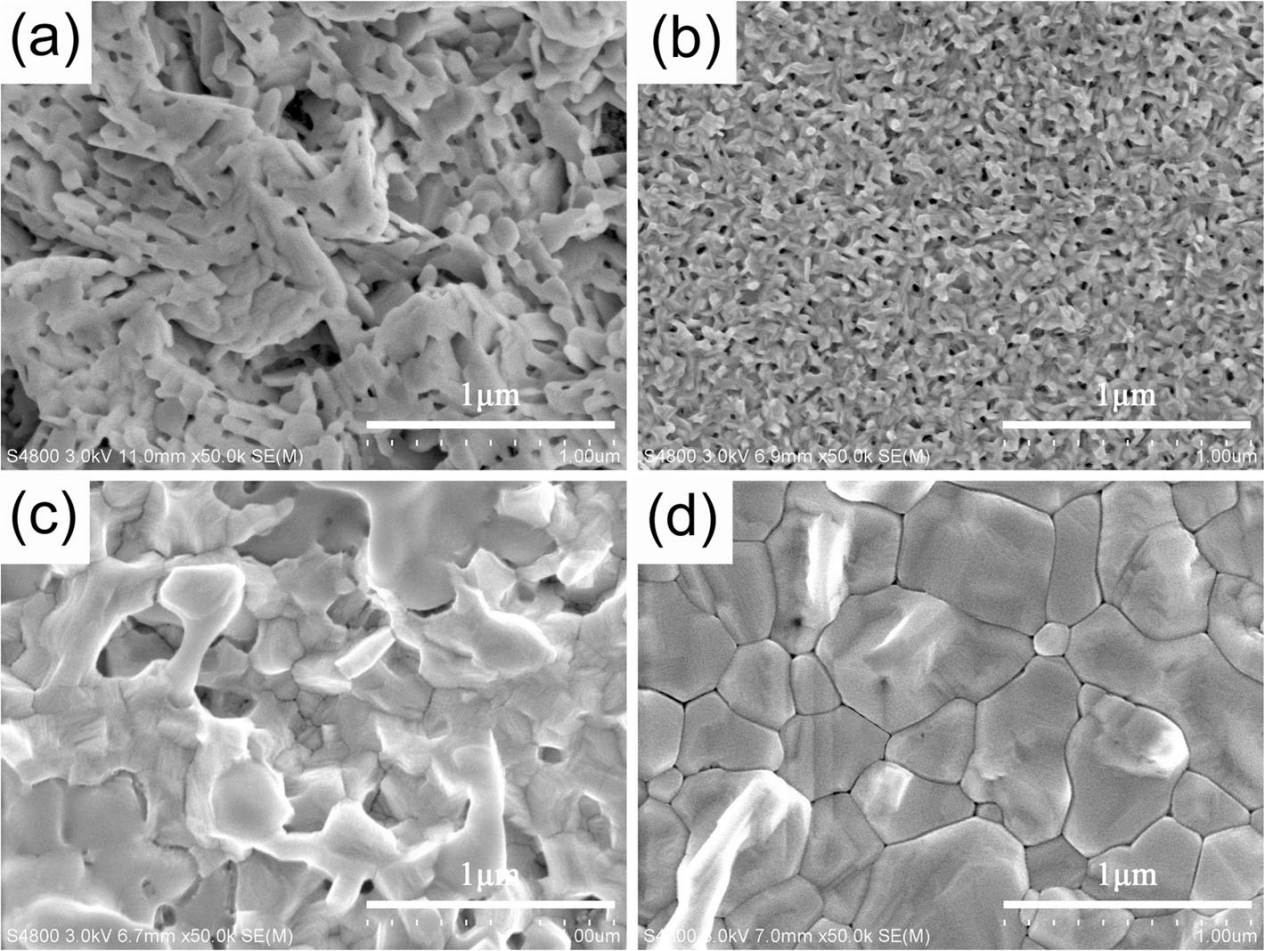
SEM images of PbI_2_ films on FTO/c-TiO_2_/mp-TiO_2_ substrates without (**a**) and with (**b**) BTA additive; and corresponding MAPbI_3_ films without (**c**) and with (**d**) BTA additive prepared under 25% RH

As known, the morphology, such as grain sizes, surface roughness, and pinholes, of perovskite films plays an important role in the performance final PSCs. For the typical two-step spin-coating process of perovskite films, controlling the morphology of PbI_2_ films is a key strategy for controlling the morphology of perovskite films [13, 19, 24]. However, it is disappointing to prepare high-quality PbI_2_ films in ambient air with 25% RH, as illustrated by the SEM image shown in Fig. 3a, which exhibits inhomogeneous and porous structure with large grain size and surface roughness. The poor quality of the PbI_2_ films under 25% RH can be mainly due to the moisture-induced rapid crystallization of PbI_2_ films. After the addition of a small amount of BTA into the PbI_2_ solution, a full coverage, continuous, and homogeneous PbI_2_ film with small grain size and low surface roughness is obtained, as presented in Fig. 3b. The high-quality PbI_2_ films can be attributed to the strong volatility, well infiltrative property, and strong Lewis base nature of BTA, who will isolate the PbI_2_ films from part of the moisture in the ambient air, help the PbI_2_ solution to spread out on the substrates homogeneously, and greatly slow down the crystallization rate to form high-quality PbI_2_ films in ambient air with 25% RH. As stated earlier, for the typical two-step spin-coating process of perovskite films, controlling the morphology of PbI_2_ films is a key strategy for controlling the morphology of perovskite films [13, 19, 24]. By virtue of the high-quality PbI_2_ films shown in Fig. 3b, high-quality MAPbI_3_ films composed of densely packed big crystal grains without any pinholes are prepared as shown in Fig. 3d, while inhomogeneous MAPbI_3_ films with small grain size and amounts of pinholes are obtained using the poor-quality PbI_2_ films as shown in Fig. 3c. In addition, the high-quality MAPbI_3_ films shown in Fig. 3d is converted from PbI_2_ in ambient air with 25% RH, indicating a small amount of moisture (25% RH) is not adverse, and even beneficial for the conversion of PbI_2_ to perovskite films.

**Fig. 4:**
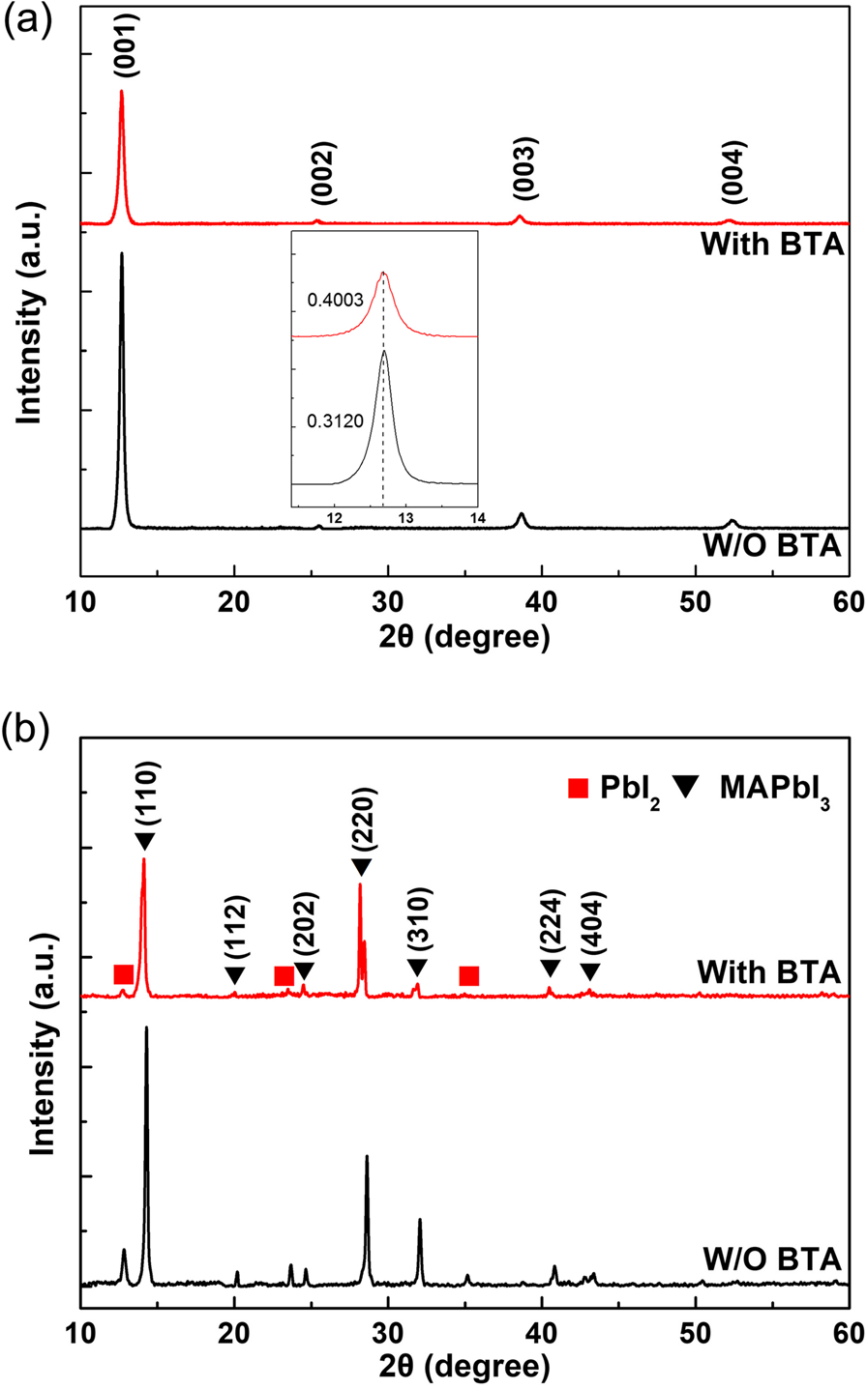
XRD spectra of PbI_2_ films (**a**) and MAPbI_3_ films (**b**) on quartz substrates prepared with and without BTA additive, in ambient air under 25% RH. The inset of (**a**) is the rocking curve of the main diffraction peak of PbI_2_ films at 12.69°

The crystal quality of as-grown PbI_2_ and MAPbI_3_ films, prepared in ambient air with and without BTA additive, was characterized by the XRD measurement. Figure 4a, b shows the XRD patterns of PbI_2_ and MAPbI_3_ films respectively, and it can be seen that the PbI_2_ and MAPbI_3_ films with and without BTA additive exhibit almost the same crystalline phase. As shown in Fig. 4a, the XRD patterns of PbI_2_ films with and without BTA additive show the intense diffraction peak at 12.69°, corresponding to the characteristic peak of PbI_2_. However, the peak at 12.69° reduces significantly in the PbI_2_ film with BTA additive, which can be explained as follows. On the one hand, as mentioned above, BTA has well infiltrative property and can help the PbI_2_ solution to spread out on the substrates easily and homogeneously. On the other hand, grain size of the PbI_2_ film with BTA additive is much smaller than the PbI_2_ film without BTA additive, as evidenced by the SEM images in Fig. 3 and the increase in full width at half maximum (FWHM) with BTA additive shown inset of Fig. 4a. Figure 3b shows the XRD patterns of MAPbI_3_ films prepared with and without BTA additive. As can be seen, the diffraction peaks were present at 2θ values of 14.06°, 20.00°, 23.45°, 28.42°, 31.86°, 40.59°, and 43.21° corresponding to the reflection planes of (110), (112), (202), (220), (310), (224), and (404) of the tetragonal perovskite structure [25], respectively. Additionally, the characteristic peak of PbI_2_, at 12.69°, is also observed in both the two MAPbI_3_ films prepared with and without BTA additive. The films prepared under ambient conditions lead to the incomplete conversion of PbI_2_ to MAPbI_3_, due to the termination of nucleation and growth of the perovskite caused by the formation of relatively continuous capping layer on the surface [26]. It is reported that a little bit of PbI_2_ can improve the performance of PSCs by passivating the defects in perovskite films [19, 26]. Furthermore, the MAPbI_3_ film prepared without BTA additive shows much higher intensity for the peak at 12.69°, compared with the film prepared with BTA additive. This suggests that there are too much PbI_2_ residual in the MAPbI_3_ film prepared without BTA additive attributed to the poor-quality PbI_2_ film without BTA additive leading to the insufficient reaction between PbI_2_ and MAI.

Combining SEM and XRD results above, it is clear what role moisture plays in the two-step spin-coating of MAPbI_3_ films in ambient air. For the PbI_2_ films, moisture can speed up the crystallizing process to form poor-quality PbI_2_ films with large grain size and surface roughness. However, for the conversion of PbI_2_ to the MAPbI_3_ films, a small amount of moisture (25% RH) is not adverse, and even beneficial.

**Fig. 5:**
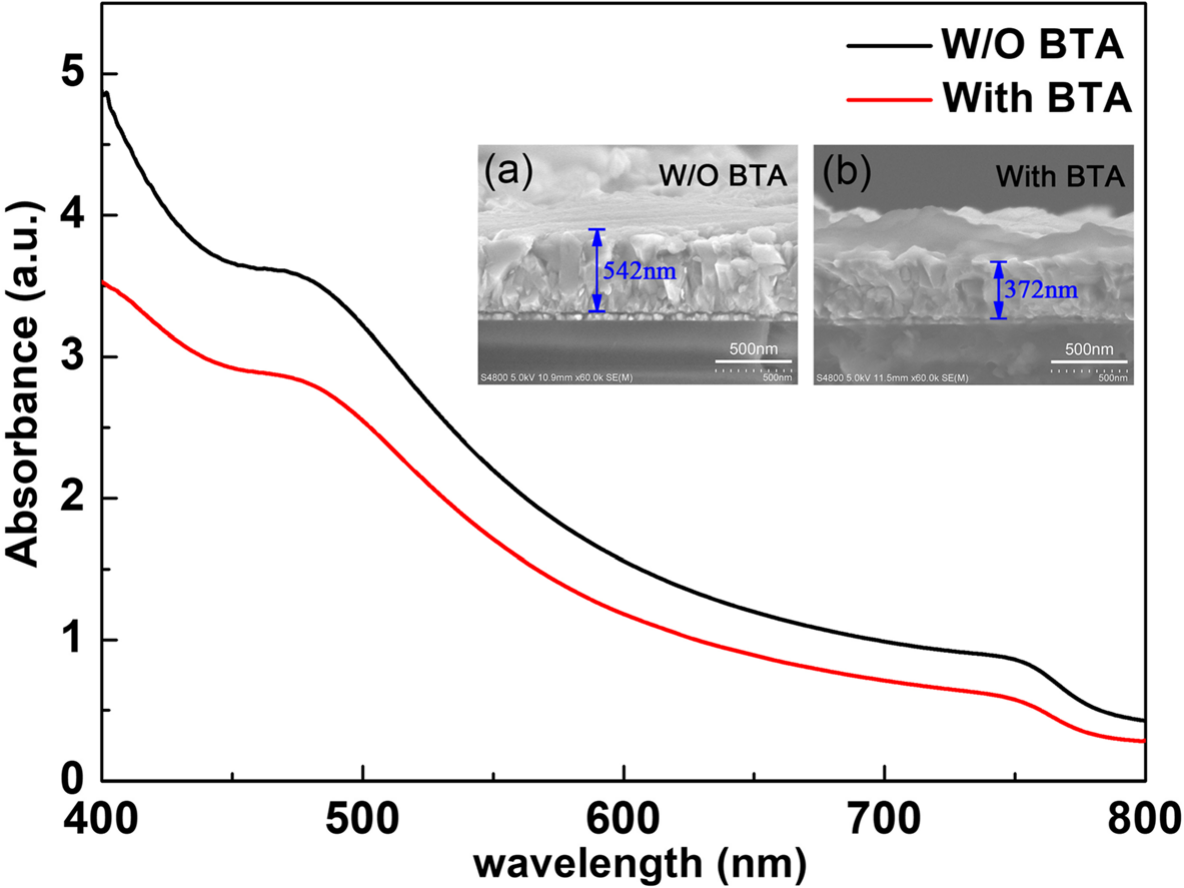
UV-Vis absorption spectrum of MAPbI_3_ films on quartz substrates with and without BTA additive. Insets are cross-section SEM images of MAPbI_3_ films prepared with (**a**) and without (**b**) BTA additive

UV-Vis absorption spectrum of MAPbI_3_ films prepared with and without BTA additive is presented in Fig. 5. All the two samples show an absorbance at a threshold of about 780 nm in the overall visible region, indicating the formation of MAPbI_3_ crystallites [27]. As can be seen, the MAPbI_3_ films with BTA additive shows lower absorbance, which is attributed to its relatively smaller thickness compared with the films without BTA additive, as confirmed in the cross-section SEM images of MAPbI_3_ films (insets of Fig. 5). Besides, the weak absorption shoulder at about 510 nm, which appears in all the two spectra, is a characteristic feature of PbI_2_, implying the residual of PbI_2_ as confirmed by the XRD measurement.

**Fig. 6:**
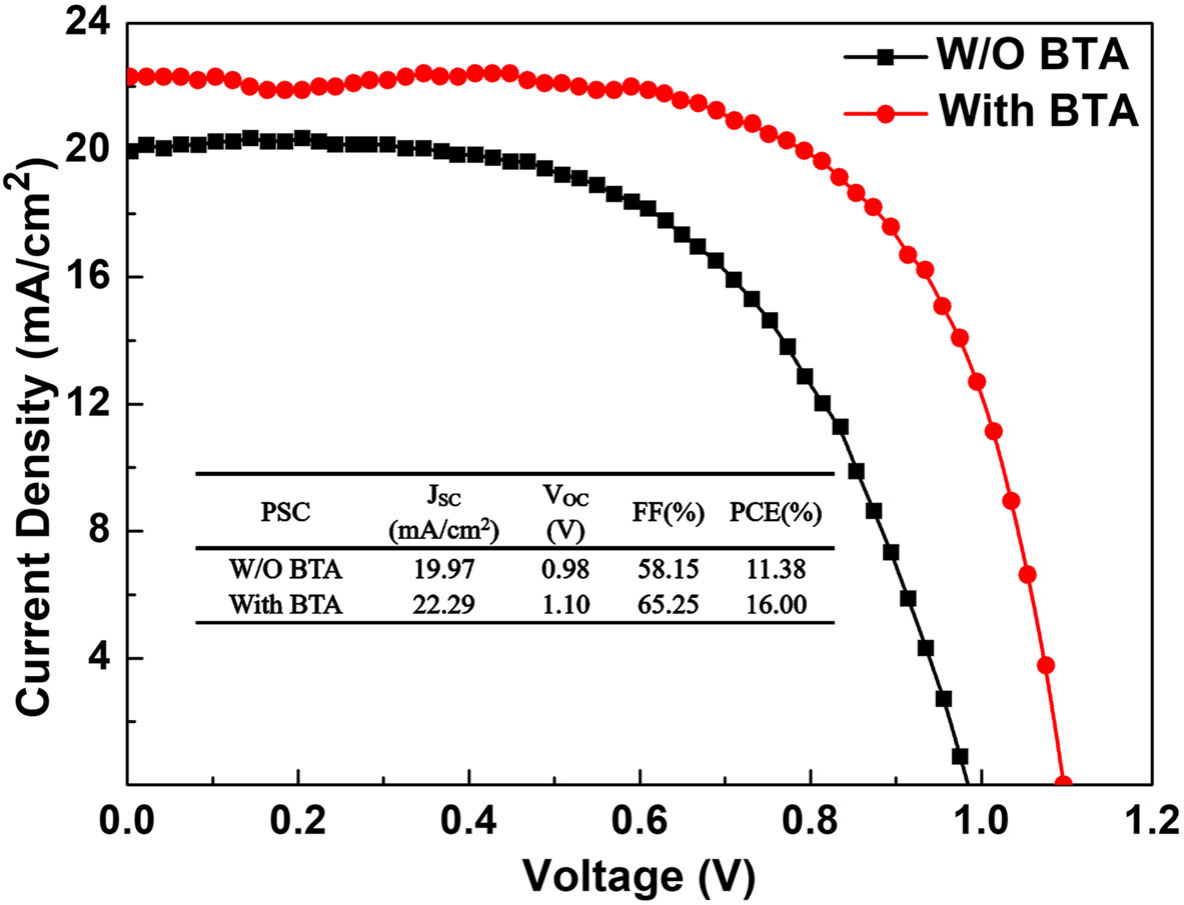
J-V characteristics of the PSCs fabricated with and without BTA additive in ambient air with 25% RH under AM 1.5G one sun (100 mW cm^−2^) illumination, the inset is the detailed photovoltaic performance

The MAPbI_3_ films prepared with and without BTA additive was then used to construct PSCs with the structure of FTO/c-TiO_2_/mp-TiO_2_/MAPbI_3_/Spiro-OMeTAD/Ag, and the corresponding J-V characteristics of the devices under AM 1.5G one sun (100 mW cm^−2^) illumination are shown in Fig. 6, the inset of which is the photovoltaic parameters. The highest PCE values in the records were adopted here for comparison. The device using perovskite films prepared without BTA additive at 25% RH showed the highest PCE of 11.38%, with J_SC_ of 19.97 mA/cm^2^, V_OC_ of 0.98 V, and FF of 58.15%. When introducing BTA additive, the devices showed significant improvements in all of the four photovoltaic parameters. There into, the device using perovskite films prepared with BTA additive showed the highest PCE of 16.00%, which is improved by ~ 40% compared with the PSCs using perovskite films prepared without BTA additive, with J_SC_ of 22.29 mA/cm^2^, V_OC_ of 1.10 V, and FF of 65.25%, which was attributed to the high-quality perovskite films with smooth surface, large crystal grains, and high crystal quality.

## Conclusions

In conclusion, we studied the effect of moisture on the formation of perovskite films in detail, and found that moisture can speed up the crystallizing process of PbI_2_ films to form poor-quality films with large grain size and surface roughness, while, for the conversion of PbI_2_ to MAPbI_3_ films, a small amount of moisture is not adverse, and even beneficial. On this basis, by adding a small amount of BTA into the solution of PbI_2_ to enhance the quality of PbI_2_ films and thus to achieve high-quality perovskite films with smooth surface, large crystal grains, and high crystal quality, we fabricated mesoporous PSCs with PCE of 16.00% under ambient air conditions at 25% RH. The results may pave a new way for fabricating efficient and reproducible PSCs under ambient air condition.

## Abbreviations

BTA: N-butyl amine
c-TiO_2_: Compact TiO_2_
DMF: N,N-dimethylformamide
FF: Fill factor
FTO: Fluorine-doped transparent conducting SnO_2_-coated glass substrates
J_SC_: Short-circuit current
J-V: Current density-voltage
Li-TFSI: Lithium bis(trifluoromethanesulfonyl)imide
MA: Methylammonium
mp-TiO_2_: MesoporousTiO_2_
PCE: Power conversion efficiency
PSCs: Perovskite solar cells
RH: Relative humidity
SEM: Scanning electron microscopy
Spiro-OMeTAD: 2,2′,7,7′-Tetrakis[N,N-di(4-methoxyphenyl)amino]-9,9′-spiro-bifluorene
TBP: 4-Tert-butylpyridine
V_OC_: Open-circuit voltage
XRD: X-ray diffraction
